# Quantitative prediction of photoluminescence quantum yields of phosphors from first principles†
†Electronic supplementary information (ESI) available: Computational details, correlation between *E*_lim_ and *x* and choosing another molecule as a reference. See DOI: 10.1039/c5sc03153b


**DOI:** 10.1039/c5sc03153b

**Published:** 2015-11-12

**Authors:** D. Escudero

**Affiliations:** a Chimie Et Interdisciplinarité , Synthèse , Analyse , Modélisation (CEISAM) , UMR CNRS no. 6320 , BP 92208 , Université de Nantes , 2, Rue de la Houssinière , 44322 Nantes Cedex 3 , France . Email: daniel.escudero@univ-Nantes.fr

## Abstract

Optimizing the photoluminescence quantum yields of Ir(iii) complexes is the key to their application as phosphors in organic light-emitting diodes (OLEDs). This work demonstrates for the first time that quantitative predictions of photoluminescence quantum yields (PLQY) in a series of blue-to-green Ir(iii) complexes can be derived exclusively from electronic structure calculations.

## Abstract 2

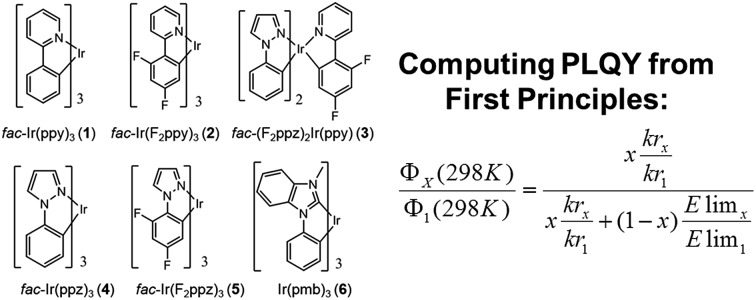
The first quantitative prediction of the photoluminescence quantum yields (PLQY) of a series of blue-to-green Ir(iii) complexes is presented.

## Introduction

Increasing academic and industrial efforts are put into the development of highly efficient electroluminescent devices. In that framework, OLEDs are excellent candidates,^1^ due to their low-cost fabrication and their exceptional electro-optical properties. Phosphorescent-based OLEDs (PhOLEDs), the so-called second generation of OLEDs, are still the most widespread devices since they can attain internal electroluminescence quantum efficiencies of almost 100%.^2^ Notably, Ir(iii) and Pt(ii) complexes are usually used as triplet emitter dopants in PhOLEDs, due to their often high internal phosphorescent efficiencies, broad range of emission colors and short excited state lifetimes.^3^ Although a wide range of Ir(iii) and Pt(ii) complexes emitting from blue to near-infrared have been reported,^3^ the number of photostable and highly efficient blue to violet complexes is still limited and their key structural–photophysical relationships are not fully understood. In that framework, recent *ab initio* and density-functional theory (DFT) studies, including spin–orbit couplings (SOCs), have provided priceless information regarding the competing deactivation mechanisms of radiative and non-radiative nature in target phosphors.^4^ Today, due to the rapid progresses in both experimental and computational techniques, we can keep track of transient states along a photodeactivation pathway and indistinguishably identify them.^5^ Their kinetic profiles of formation and decay can be followed as well. Hence, a fully detailed understanding of the fate of excited phosphors usually requires the synergy of experiments and calculations. The recent improvements in theoretical methods have extended the applications from a qualitative assignment of the absorption and emission color to a quantitative interpretation of both photochemical reactivity and emission spectroscopy.^6^ Still, the theoretical estimation of PLQY remains difficult, due to the intricate nature of the competing deactivation processes, which are often temperature-dependent. The accurate estimation of PLQY, a central experimental quantity, would be extremely beneficial for the *in silico* prescreening of promising OLED materials. In this contribution, I present for the first time a quantitative estimation of PLQY of a series of blue-to-green Ir(iii) emitters exclusively based on electronic structure calculations and the use of simplified kinetic models.

**Chart 1:**
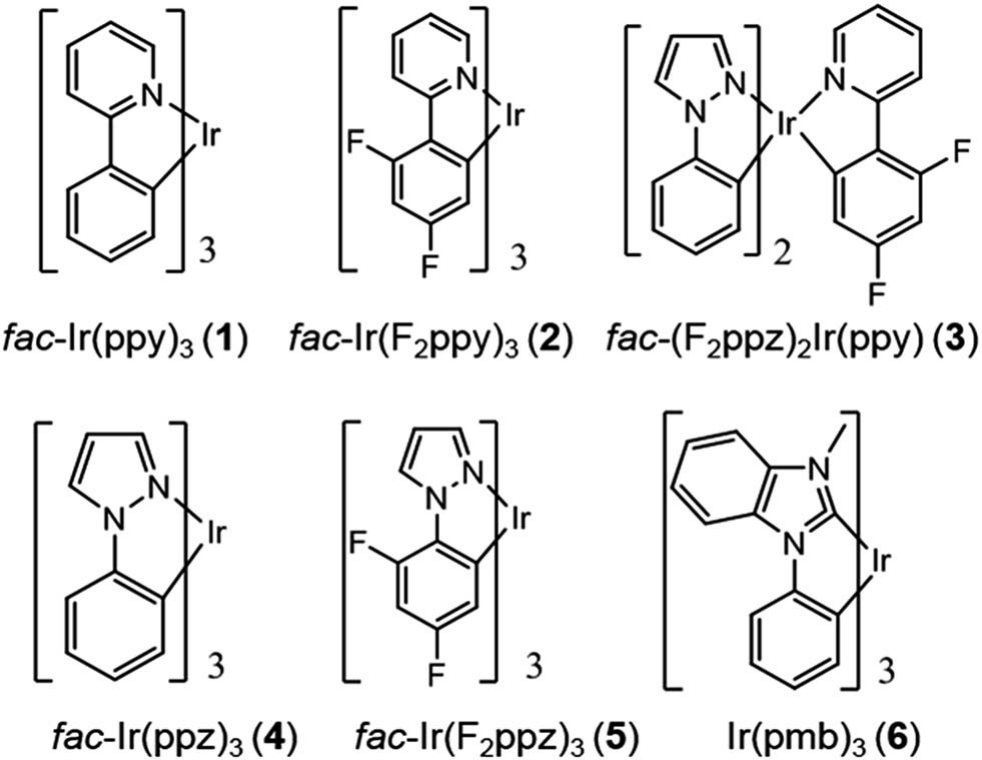
Chemical structure of complexes **1–6**.

Chart 1 gathers the homoleptic and heteroleptic Ir(iii) complexes studied here, which include common strategies to attain blue phosphorescence, *e.g.* (i) addition of fluorine to the phenylpyridine (ppy) ligand, **2**; (ii) use of other cyclometalating ligands attaining high triplet energies, such as phenylpyrazole (ppz), **3–5** and (iii) use of N-heterocyclic carbene ligands, such as the 1-phenyl-3-methylbenzimidazolyl (pmb) ligand, **6**. These phosphors exhibit short radiative emissive decay times (that is, large radiative rates), which is beneficial both to attain high PLQY and to reduce the undesired roll-off effects originated from triplet–triplet annihilation (TTA) processes. The spectroscopic properties of **1–6** have been exhaustively investigated by Thompson and coworkers.^7^ A special focus was put on (i) the rigorous determination of their PLQY and (ii) the interpretation of the temperature-dependent photoluminescence data. Besides, femtosecond transient-absorption experiments on pseudo-octahedral Ir(iii) complexes have shown that after excitation of the manifold of singlet excited states, ultrafast intersystem crossing (ISC) occurs in less than 100 fs in a “horizontal” manner,^8^ leading to the formation of the triplet states with near-unity quantum yield, and hence determining that relaxation processes are dominated by decay of the triplet excited states. These ultrafast relaxation processes are based on the proper energetic alignment between the singlet and triplet metal-to-ligand charge transfer (MLCT) excited states, which are efficiently coupled *via* large spin–orbit couplings (SOCs). Therefore, in these complexes, the emission usually takes place from the lowest triplet excited state, *i.e.* the Kasha state, although emission from higher-lying states has been reported for some complexes.^9^ In order to ensure efficient phosphorescence, a large T_1_ → S_0_ SOC value is required. In practice, the larger the MLCT character of the emissive state, the more efficient the radiative process. The phosphorescence radiative decay rate constants (*kir*) from one of the three spin sublevels (indexed by *i*) of the involved emissive state (T_1_) can be expressed as^10^1
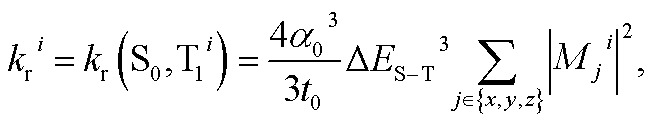
where Δ*E*_S–T_ is the transition energy, *α*_0_ is the fine-structure constant, *t*_0_ = (4π*ε*_0_)^2^/*m*_e_*e*^4^ and *M*_*j*_^*i*^ is the *j* axis projection of the electric dipole transition moment between the ground state and the *i*^th^ sublevel of the emissive triplet state, T_1_. At room temperature (RT), only weighted phosphorescence rates can be measured. Accordingly, phosphorescence rates are:2
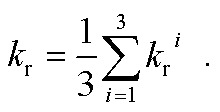



## Results and discussion

In Table 1 the photophysical properties of complexes **1–6** are listed. The radiative rates have been computed with quadratic response (QR) time-dependent (TD) DFT calculations^11^ (see Computational details in the ESI†).

**Table 1:** Photophysical data of complexes **1–6**

Complex	*k*_r_ (exp RT, s^–1^)^*a*^	*k*_r_ (theo, s^–1^)	*k*_r*x*_/*k*_r1_(exp), *k*_r*x*_/*k*_r1_*(theo)*^*b*^	*Φ*_P_ (exp, RT)^*a*^ *Φ*_P_*(theo)*^*b*^
**1**	6.1 × 10^5^	1.1 × 10^5^	—	0.97 *(—)*
**2**	5.8 × 10^5^	9.8 × 10^4^	0.95 *(0.89)*	0.98 *(0.88)*
**3**	4.6 × 10^5^	1.1 × 10^5^	0.75 *(1.00)*	0.55 *(0.63)*
**4**	—^*c*^	1.9 × 10^4^	— *(0.17)*	<0.01 *(0.16)*
**5**	—^*c*^	1.1 × 10^4^	— *(0.10)*	<0.01 *(0.11)*
**6**	3.4 × 10^5^	2.4 × 10^4^	0.56 *(0.22)*	0.37 *(0.57)*

^*a*^From ref. 7 in 2-MeTHF.

^*b*^Theoretical estimates are presented in parentheses.

^*c*^The experimental radiative rates could not be determined.

**Scheme 1:**
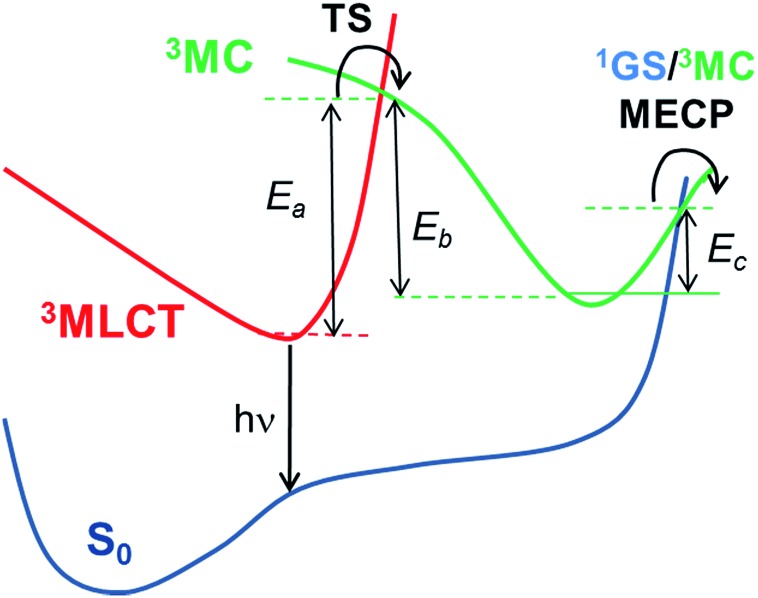
Schematic representation of the temperature-dependent non-radiative channels of Ir(iii) complexes.

The QR TD-DFT approach has proven successful for other Ir(iii) complexes^12^ and typically gives *k*_r_ values which are systematically slightly underestimated with respect to the experimental values, as previously described for other perturbative approaches.^13^ Still, the ratio between radiative rates (using **1** as reference, *i.e. k*_r*x*_/*k*_r1_) reasonably reproduces the experimental ones (see Table 1). Hence, **1–3** possess the larger *k*_r_ values both experimentally and theoretically whereas **4** and **5** possess *ca.* one order of magnitude smaller values (note that the experimental rates could not be determined due to their negligible *Φ*_P_ values). Finally, an intermediate *k*_r_ value is obtained for **6**. As seen in Table 1, there is a certain degree of correlation between the *k*_r*x*_/*k*_r1_ ratio and the *Φ*_P_ values. Obviously, larger *k*_r_ values lead to increased photoluminescence efficiencies. However, if only the radiative rates are considered, one can not rationalize all the experimental trends, *e.g.* one cannot explain why, despite its large *k*_r_ value, **3** attains smaller PLQY than **1** and **2**. Obviously, non-radiative mechanisms are responsible for these discrepancies. Hence, the radiative efficiency is not the only factor controlling the PLQY. The PLQY, *i.e. Φ*_Phos_(*T*), can be expressed as,3
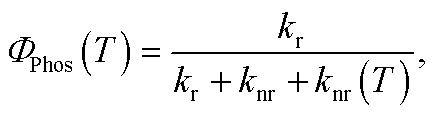
and depends on: (i) the radiative rate (*k*_r_), which is assumed to be temperature-independent provided that the three substrates of the lowest triplet excited state are equilibrated and other thermally activated emissive states are not populated; (ii) the non-radiative temperature-independent decay rate (*k*_nr_), which is associated with the overlap between the S_0_ and T_em_ vibrational wave functions and follows the energy gap law;^14^ and (iii) the strongly temperature-dependent non-radiative rate, *k*_nr_(*T*), which is connected to the thermal population of a non-radiative excited state. Given the high *k*_r_ values for these complexes, the principal mechanism that promotes nonradiative decay in green-to-blue phosphors is the temperature-dependent one, *i.e. k*_nr_(*T*).^7^ In contrast, *k*_nr_ are generally two orders of magnitude smaller than *k*_r_ and they can consequently be neglected during the computation of PLQY. The PLQY are strongly temperature-dependent, since all the complexes are highly emissive at 77 K (*Φ*_Phos_ ≈ 1) but not at 298 K (see Table 1).^7^ As OLEDs should work at ambient temperatures, controlling the temperature-dependent behavior is vital for designing more efficient phosphors. Computational studies have provided very important insights into the temperature-dependent non-radiative photodeactivation pathways of pseudo-octahedral Ir(iii) complexes,^15^ but also of square-planar Pt(ii) complexes.^16^ These studies confirmed the active role of metal centered (^3^MC) triplet excited states in these pathways. Hence, as schematically represented in Scheme 1, pseudo-octahedral Ir(iii) complexes at their T_1_ geometry (with a predominant ^3^MLCT character) usually need to surpass a barrier (see the transition state, *i.e.* TS, in Scheme 1) to populate the geometry of the lowest ^3^MC state (which commonly displays a trigonal bipyramid arrangement). Once the ^3^MC well is populated, two main processes may follow: (i) reversible return to the ^3^MLCT well; or (ii) irreversible recovery of the ground state (^1^GS) geometry.^4^ The energy barrier of the latter process is determined by the ^1^GS/^3^MC minimum energy crossing point (MECP). The MECP geometry usually exhibits a further distorted trigonal bipyramid arrangement. This kinetic scenario can be summarized as;4
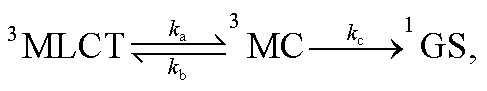
where *k*_a_, *k*_b_ and *k*_c_ are the kinetic rates of the temperature-dependent non-radiative channels. The temperature-dependent non-radiative rate in eqn (3), *i.e. k*_nr_(*T*), can be expressed using a Boltzmann model,

5 *k*_nr_(*T*) = *A*exp(–*E*_lim_/*k*_Boltz_*T*),

where *E*_lim_ is the activation energy for the limiting step and *k*_Boltz_ is the Boltzmann constant.^4^ To characterize these pathways and to optimize the ground and lowest triplet excited states along the photodeactivation coordinate, DFT calculations are often used.^15^ DFT succeeds in reaching a continuous adiabatic description of these excited state potential energy surfaces (PES).

Two kinetic scenarios can be found. The first scenario arises when the formation of the ^3^MC state is the rate limiting (*i.e.* rate determining) step (*i.e. E*_a_ is the kinetic bottleneck, see Scheme 1). Thus, large values of *E*_a_ (*i.e. E*_a_ ≫ *E*_c_) lead to the efficient quench of the temperature-dependent non-radiative channels. The second kinetic scenario arises when the MECP barrier is the rate limiting step (*i.e. E*_c_ ≫ *E*_a_). This latter scenario is less beneficial for improving the PLQY. Finally, in the former scenario (*E*_a_ ≫ *E*_c_) two possible subkinetic cases can be distinguished: (a) if *E*_b_ ≫ *E*_c_, upon population of the ^3^MC well, the back reaction will not be favoured, so that the complex will efficiently undergo irreversible intersystem crossing to the ^1^GS PES; (b) if *E*_c_ ≥ *E*_b_, the barrier for the back reaction has been lowered, so that a pre-equilibrated ^3^MLCT–^3^MC situation is reached. Hence, the return to the ^3^MLCT state is at least as favoured as the irreversible recovery of the ^1^GS geometry.

**Fig. 1:**
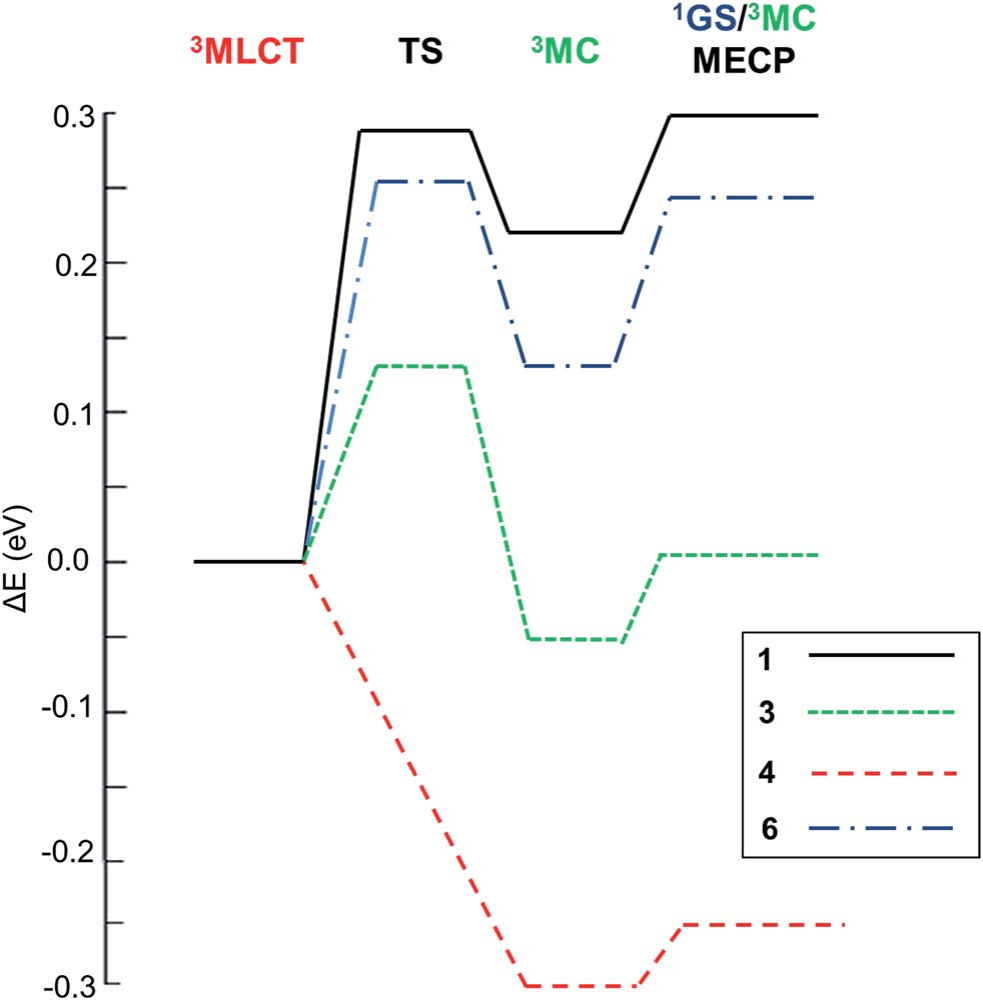
Relative energetic profile (B3LYP/6-31G(d)) of the temperature-dependent non-radiative pathways of **1–6**. The reference is the ^3^MLCT emissive state.

With this kinetic model in mind, the barriers of the rate-limiting process were evaluated, *i.e. E*_lim_ (see Table 2), which is an estimator of the efficiency of the temperature-dependent quenching of photoluminescence. To obtain the values of *E*_a_, *E*_b_ and *E*_c_ in Scheme 1, the geometries of the ^3^MLCT and ^3^MC states, of the TS and of the ^1^GS/^3^MC MECP stationary points of **1–6** were optimized using the B3LYP functional (see the Computational details in the ESI†). Next, their relative energies were evaluated. Key data are collected in Table 2. The energy profiles of selected complexes along the deactivation coordinate are shown in Fig. 1. In this series, **1** and **2** follow the *E*_a_ ≫ *E*_c_ kinetic scenario and they possess the largest *E*_lim_ values (*ca.* 0.3 eV, see for example the energetic profile of **1** in Fig. 1). As experimentally corroborated,^7^ these barriers are large enough to prevent the population of these non-radiative channels at RT (they only become operative at temperatures exceeding 300 K). In **4** and **5** (see **4** in Fig. 1) the emissive ^3^MLCT state is adiabatically located higher in energy than the ^3^MC state. The ^3^MC well is accessed in a barrierless manner, since no TS is found along the ^3^MLCT → ^3^MC reaction coordinate. There is a small barrier to populate the ^1^GS/^3^MC MECP geometry (*ca.* 0.05 eV, see Table 2), which is the rate limiting step. Having in mind the small *E*_lim_ values in **4** and **5**, thermally-activated decay is highly efficient even below room temperature. Indeed, their experimental lifetimes hugely decrease from 150 K to 200 K, leading to a complete quench of photoluminescence at RT (*Φ*_Phos_ < 0.01).^7^ Hence, **4** and **5** are the complexes most prone to non-radiative deactivation. Finally, for the carbene complex **6** and the heteroleptic complex **3** the formation of the ^3^MC state is the rate limiting step as in **1** and **2**. By comparing their relative *E*_b_ and *E*_c_ energies they can be classified as pre-equilibrated (**6**) or not (**3**), see their energetic profiles in Fig. 1 and values in Table 2.

**Table 2:** Activation barriers (eV) for the temperature-dependent non-radiative channels (see Scheme 1) and prefactor *x* for **1–6**

Complex	*E*_a_	*E*_b_	*E*_c_	*E*_lim_ ^*a*^	*x*
**1**	0.287	0.064	0.075	0.298^*b*^	1
**2**	0.272	0.096	0.077	0.272	0.91
**3**	0.136	0.200	0.067	0.136	0.46
**4**	0.000	0.307	0.042	0.042	0.14
**5**	0.000	0.348	0.060	0.060	0.20
**6**	0.252	0.118	0.088	0.252	0.85

^*a*^The *E*_lim_ value usually corresponds to *E*_a_ or *E*_c_ value, depending on the kinetic scenario.

^*b*^For **1**, since the MECP barrier is above the TS barrier, *E*_lim_ is obtained according to *E*_lim_ = *E*_a_ + *E*_c_ – *E*_b_.

With the computed (i) radiative rates and (ii) energy barriers (*E*_lim_) I propose to use the following simplified expression to compute the relative PLQY at 298 K of any of the complexes, *i.e. Φ*_*X*_(298 K), with respect to **1** at 298 K, *i.e. Φ*_1_(298 K) = 0.97:6
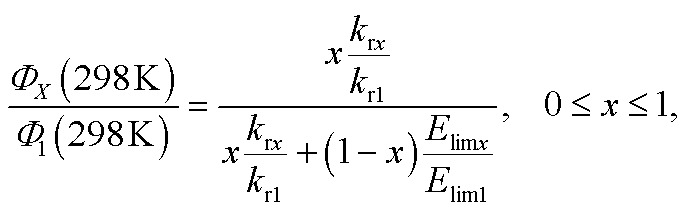



**Chart 2:**
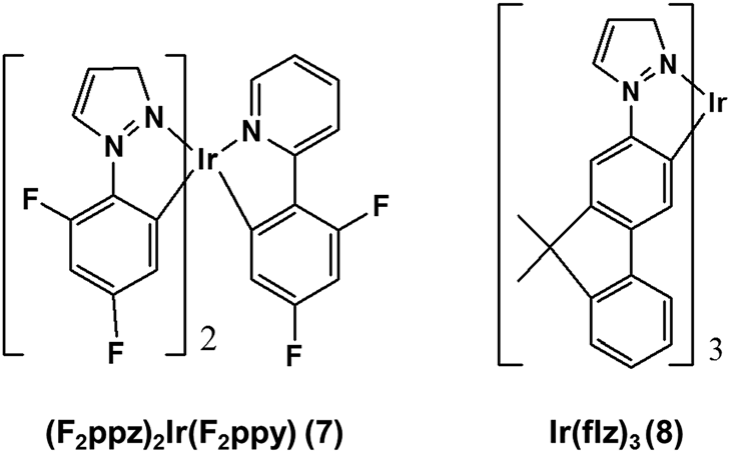
Chemical structure of the validator complexes **7** and **8**.

The form of eqn (6) resembles that of eqn (3), provided that the temperature-independent non-radiative decay rates (*k*_nr_) are neglected at RT, which is a reasonable assumption for green-to-blue phosphors, as corroborated experimentally.^7^Eqn (6) further introduces *x*, which is a scaling prefactor of order unity determining the availability of the temperature-dependent non-radiative channels at RT, which are mainly dependent on the *E*_lim_ values at a given temperature (see eqn (5)). In the following I analyze in depth the emissive properties of **1–6** to develop realistic models of the PLQY, which in practical terms means reaching appropriate estimations of the *x* scaling prefactors introduced in eqn (6). For **1** and **2**, the temperature-dependent non-radiative channels are negligible, since they possess PLQY of almost unity (see the *Φ*_Phos_ = 0.97–0.98 values in Table 1). Therefore, when estimating their PLQY values with eqn (6), the *k*_r*x*_/*k*_r1_ ratio is the only factor determining their PLQY. I note that their computed *E*_lim_ values are the largest among all the complexes. In contrast, for **3–6**, since their experimental PLQY are clearly smaller than the unity of quantum yield, the *E*_lim*x*_/*E*_lim1_ factor should be concomitantly evaluated with the *k*_r*x*_/*k*_r1_ ratio, *i.e.* the non-radiative pathways are fully activated at RT for these complexes. The fact that the *E*_lim_ values for **3–6** are smaller as compared to those for **1** and **2** clearly indicates that there is a correlation between the *E*_lim_ and *x* values. Indeed, **4** and **5**, the non-emissive complexes at RT, are characterized by the lowest *E*_lim_ values amongst all the complexes. Gathering all this information, to obtain the *x* values in eqn (6) one further needs to assess (i) what type of correlation between the *E*_lim_ and *x* values is more appropriate (*i.e.*, linear or non-linear) and (ii) which are the limit conditions in the correlation fit. Different correlation models between the *E*_lim_ and *x* values were evaluated, from linear correlation models (see models 1–2 in Section 2 of the ESI†) to non-linear models (see the hyperbolic model 3 in Section 2 of the ESI†). The effect of changing the limit conditions in the models was also evaluated (compare model 1, with *x* = 1 → *E*_lim1_ = 0.298 eV and *x* = 0 → *E*_lim_ = 0 as limit conditions, to model 2, with *x* = 1 → *E*_lim1_ = 0.298 eV and *x* = 0 → *E*_lim4_ = 0.042 eV as limit conditions, see the ESI†). The well-known experimental facts for **1–6** guide the construction of these models (see the specific details for each model in the ESI†). The *x* prefactors for complexes **1–6** using model 1, which are obtained by extrapolating the *E*_lim_ values on the linear correlation fit shown in Fig. S1,† are shown in Table 2 along with the estimated *Φ*_*X*_(298 K) values in Table 1. The summary of the results using all possible models is presented in Table S3.† The estimated PLQY show a good quantitative agreement with respect to their experimental counterparts, regardless of the model used. Hence, eqn (6) is able to discern from highly emissive complexes at RT, *e.g.***2**, to complexes almost non-emissive, *e.g.***4** and **5** or complexes with intermediate PLQY values, *e.g.***3** and **6**. Furthermore, the computed PLQY values exhibit the same trend as the experimental ones, *i.e.***2** > **3** > **6** > **4** ≥ **5**, except in the case of model 3, which reverses the order of complexes **3** and **6** (see Tables S2 and 3†). This deserves further exploration. An in depth analysis of **6** reveals that, regardless of the model used, the computed PLQY values are overestimated with respect to the experimental one (see Table S3†). This likely originates from its pre-equilibrated ^3^MLCT–^3^MC scenario (see discussion above), and thus eqn (6), which only considers the barrier of the rate-determining step, does not fully restore the kinetic complexity of the photodeactivation processes occurring in **6**. The effect of choosing a different reference molecule, *i.e.***2** instead of **1**, to compute the PLQY with eqn (6) has also been assessed. These results are presented in Section 3 of the ESI. † As seen in Table S4,† the results are not affected by choosing a different reference molecule. Therefore, to keep consistency with the experimental data,^7^ I recommend the use of **1** as a reference molecule. To sum up, the choice of the model (models 1–3) has an influence on the PLQY results but it does not have a great impact on the qualitative pre-screening of phosphors. On the contrary, the results appear to be insensitive to choosing a different reference molecule. Next, to further corroborate the validity of the models to compute the PLQY I now proceed to evaluate two other Ir(iii) complexes that did not participate in the construction of the models. Thus, **7** and **8** (see Chart 2) are used herein as external validators. Complex **7** is a new heteroleptic complex whilst **8** is a new homoleptic complex bearing a different ligand scaffold from **1–6**, *i.e.* the 1-1-(2-(9,9′-dimethylfluorenyl))pyrazolyl (flz) ligand. Their experimental emissive properties from ref. 7 are listed in Table 3. Their radiative rates and the PES of the temperature-dependent non-radiative deactivation pathways were obtained using the same computational protocol as for **1–6**. Key computed data are collected in Tables 3 and S5.† Their estimated PLQY values using eqn (6) and model 1 are also tabulated in Table 3. The PLQY values with models 2 and 3 can be found in Table S5.† For both complexes the population of the ^3^MC state is the rate determining step (see Table S5†). In general, the results for **7** and **8** do not heavily depend on the model used. As seen in Table 3, the estimated PLQY agree reasonably well with the experimental ones. This is also the case for **8**, which despite its very small *k*_r_ value still retains a very large PLQY at RT. Thus, eqn (6) succeeds in predicting the PLQY with a reasonable accuracy in a wide variety of kinetic scenarios. The large PLQY in **8** can be understood in terms of its very large *E*_lim_ value (*i.e.* 0.311 eV, see Table S5†), which makes the temperature-dependent non-radiative pathways not accessible at RT. The small deviation between the experimental and estimated PLQY value in **8** likely originates from the neglect of the temperature-independent non-radiative pathways in eqn (6), which become more important in **8** than in **1–7** due to its considerably decreased *k*_r_ value. Still, eqn (6) is capable of discerning between a highly emissive complex (**8**) and an intermediately emissive one (**7**).

**Table 3:** Photophysical data of complexes **7** and **8**

Complex	*k*_r_ (exp RT, s^–1^)^*a*^	*k*_r_ (theo, s^–1^)	*k*_r*x*_/*k*_r1_(exp) *k*_r*x*_/*k*_r1_*(theo)*^*b*^	*Φ*_P_ (exp, RT)^*a*^ *Φ*_P_*(theo)*^*b*^
**7**	4.6 × 10^5^	6.0 × 10^4^	0.75 *(0.55)*	0.60 *(0.63)*
**8**	1.7 × 10^4^	4.5 × 10^3^	0.03 *(0.04)*	0.81 *(0.97)*

^*a*^From ref. 7 in 2-MeTHF.

^*b*^Theoretical estimates are presented in parentheses.

In a nutshell, the use of eqn (6) as a pre-screening strategy of promising green-to-blue Ir(iii) complexes for OLEDs applications is demonstrated. The results appear to be robust with regard to the simplified kinetic models used and the considerations taken in the construction of eqn (6). I remark that, to my knowledge, this is the first reported approach to compute the PLQY of phosphors. Still, it is important to remark the limitations of the present approach to compute PLQY, *i.e.* (i) the simplified kinetic model (which only considers the barrier of the rate determining step), (ii) the neglect of the temperature-independent non-radiative pathways, (iii) the assumption that the ISC processes are the unity of quantum yield, and (iv) the considerations taken in the construction of eqn (6). Whilst (iii) generally remains valid for Ir(iii) complexes and (iv) is considerably validated with the different models proposed herein, the two former points require further discussion. Hence, eqn (6) should be used with caution in pre-equilibrated ^3^MLCT–^3^MC scenarios, as shown for complex **6**. It should also be used with caution in cases where the dominating non-radiative processes are the temperature-independent ones, *i.e.*, those arising from the overlap between vibrational wave functions, which follow the energy gap law. Therefore, eqn (6) might not be appropriate for red to near infrared (NIR) Ir(iii) complexes, since their red-shifted transition energies, *i.e.* Δ*E*_S–T_, lead to predominance of these pathways. In the case of blue-to-green phosphors, as reported herein, eqn (6) remains valid for a large diversity of heteroleptic and homoleptic complexes bearing different ligand scaffolds, including carbene ligands. Thus, it can presumably be used in a general way.

## Conclusions

In this paper I present the first theoretical approach to quantitatively estimate the PLQY of blue-to-green phosphor molecules. Several models to compute the PLQY have been tested. The results obtained on the initial set of molecules (**1–6**) and on the external validators (**7** and **8**) demonstrate that these simplified kinetic models are robust yet simple approaches to compute PLQY. To obtain the PLQY only a few calculations are needed, *i.e.* computing radiative rates from the emissive state and characterizing the PES of the temperature-dependent non-radiative deactivation channels. As in the experimental setups, a reference value is needed, which in this work is the experimental *Φ*_Phos_(298 K) value of complex **1**. Future work will be devoted to developing more complex PLQY estimators, also applicable for red-to-NIR complexes. I remark that the latter complexes may require further progress from a theoretical viewpoint, since they will require the concomitant calculation of the temperature-independent non-radiative rates.

## Supplementary Material

Supplementary information
