# Supplementary material for: Quantitative prediction of photoluminescence quantum yields of phosphors from first principles
Source: Chem Sci. 2015 Nov 12;7(2):1262–7. doi: 10.1039/c5sc03153b (PMC5975840; doi:10.1039/c5sc03153b)
Supplement: Supplementary file 1 [file SC-007-C5SC03153B-s001.pdf]

Electronic Supplementary Material (ESI) for Chemical Science.
This journal is © The Royal Society of Chemistry 2015

Supporting Information

Quantitative Prediction of Photoluminescence Quantum Yields of Phosphors from First Principles.

Daniel Escudero,[a,*]

[a] Chimie Et Interdisciplinarité, Synthèse, Analyse, Modélisation (CEISAM), UMR CNRS no. 6320, BP 92208, Université de Nantes, 2, Rue de la Houssinière, 44322 Nantes, Cedex 3, France.

1. Computational Details page 2-3
2. Correlation between E_{lim} and x page 4-8
3. Choosing another molecule as a reference page 9
4. Table S5 page 10
5. References page 11

1. Computational details

All calculations are based on density functional theory (DFT). The geometries of the singlet ground state (^{1}GS), the lowest triplet excited state, and the transition states (TS_1) were optimized for complexes **1-6** using the hybrid functional B3LYP[1,2] in combination with the 6-31G* basis set for all atoms. Relativistic effects were included for the Ir atom by using the ECP-60-mwb Stuttgart/Dresden pseudopotential.[3] The nature of the stationary points was confirmed by computing the Hessian at the same level of theory. The minimum energy crossing point (MECP) between the ^{1}GS and the ^{3}MC potential surfaces was optimized using Harvey´s algorithm,[4] as implemented in the ORCA software;[5] in this case, the B3LYP functional was employed in combination with the def2-svp basis set and the ECP-60-mwb Stuttgart/Dresden pseudopotential for Ir. To get relative energies for the MECP, single-point calculations with the 6-31G* basis set were performed. All calculations apart from the MECP optimization were carried out with the Gaussian09 program package.[6]

The phosphorescence radiative decay rates were computed using the QR TD-B3LYP approach, as implemented in the Dalton program, [7] at the optimized geometry of the emissive triplet state (T_{em}) of complexes **1-6**. The rate constants (k_r) for phosphorescence radiative decay from one of the three spin sublevels (indexed by i) of the emissive states (T_m) can be expressed as

$$k_r^i = k_r\left(S_0, T_{em}^i\right) = \frac{4\alpha_0^3}{3t_0}\Delta E_{S-T}{}^3 \sum_{j\in\{x,y,z\}} \left|M_j^i\right|^2 \quad (1),$$

where $\Delta E_{S\text{-}T}$ is the transition energy, $t_0 = (4\pi\varepsilon_0)^2/m_e e^4$, α_0 is the fine-structure constant, and M_j^i is the j axis projection of the electric dipole transition moment between the ground state and the ith sublevel of the triplet state T_{em}. The transition moment M_j^i can be expressed as,

$$M_j^i = \sum_{n=0}^{\infty} \frac{\langle S_0|\hat{\mu}_j|S_n\rangle\langle S_n|\hat{H}_{SO}|T_{em}^i\rangle}{E(S_n) - E(T_{em})} + \sum_{n=0}^{\infty} \frac{\langle S_0|\hat{H}_{SO}|T_n\rangle\langle T_n|\hat{\mu}_j|T_{em}^i\rangle}{E(T_n) - E(S_0)}, j \in \{x, y, z\} \quad (2),$$

which is calculated using the QR TD-B3LYP approach.[8] Note that individual phosphorescence rates for the three spin sublevels can only be observed experimentally in the limit of large fine-structure splittings

and at low temperatures. In the high-temperature limit, spin relaxation is usually fast and the triplet levels are almost equally populated, and only weighted phosphorescence rates can be measured. Hence, phosphorescence rates are calculated according to (3).

$$k_r = \frac{1}{3}\sum_{i=1}^{3} k_r^i \tag{3}$$

In the QR TD-B3LYP calculations the 6-31G and raf-r basis set were used for light atoms and Ir, respectively. Scalar relativistic effects were included with the Douglas-Kroll-Hess second order (DKH2) Hamiltonian.[9] The SOC operator applied in all our calculations makes use of a semi-empirical effective single-electron approximation, as suggested by Koseki et al.[10]

2. Correlation between E_{lim} and x

In Eq(6) of the manuscript,

$$\frac{\Phi_X(298K)}{\Phi_1(298K)} = \frac{x\frac{kr_x}{kr_1}}{x\frac{kr_x}{kr_1} + (1-x)\frac{E\lim_x}{E\lim_1}}, \qquad 0 \le x \le 1 \qquad (6),$$

x is introduced, which is a scaling prefactor of order unity determining the availability or not of the temperature-dependent non-radiative channels at RT. For complexes **1**-**2**, these channels are negligible, since they possess PLQY values of almost unity at RT (see the $\Phi_{phos} = 0.97–0.98$ values in Table 1 of the manuscript). The computed *Elim* values for **1**-**2** are the largest ones among all the complexes. Therefore, since the non-radiative pathways are not operative at this temperature, when estimating their PLQY values with Eq. (6), only the kr_x/kr_1 ratio determines the PLQY. In contrast, for complexes **3**-**6**, since their PLQY are clearly smaller than the unity of quantum yield (i.e. the non-radiative pathways are fully operative at RT), the $Elim_x/Elim_1$ factor in Eq. (6) should be concomitantly evaluated with the kr_x/kr_1 ratio. Note that the *Elim* values for **3-6** are smaller as compared to **1**-**2**. These evidences show that there is a clear correlation between the *Elim* and the *x* values. To define such a correlation one needs of two further assumptions, namely i) which are the limit conditions in the correlation fit and ii) what type of correlation (e.g. linear, hyperbolic, etc.) is more appropriate. In the following I explore the robustness of the model by deeply exploring the correlation between the E_{lim} and x values.

Linear correlation between the *Elim* and the *x* values (Model 1): This is the original model presented in the manuscript (Model 1). A linear correlation between the x and E_{lim} values is considered, assuming the following limit conditions: x=1→ $Elim_1$=0.298 eV (note that this is the computed *Elim* value for complex 1) and x=0→ *Elim*=0 eV. Complexes with *Elim* values above the upper limit condition (i.e., *Elim*>0.298 eV) possess x=1. These limit conditions assume that the non-radiative pathways are fully quenched for complex 1 at RT (only the kr_x/kr_1 ratio determines the PLQY values in Eq.(6)), whilst in the other extreme case (x=0) these processes occur in a barrierless manner and thus, only the $Elim_x/Elim_1$ ratio dominates in Eq. (6). This correlation fit is shown in Fig S1. The x values tabulated in Table 2 of the manuscript for complexes **2-6** are extrapolated from this correlation fit.

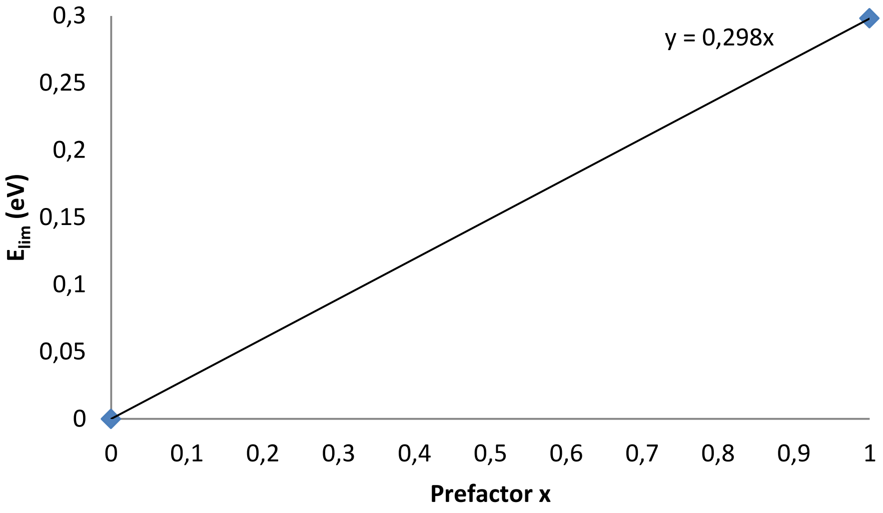

Figure **S1.** Linear correlation between the E_{lim} and x values using as limit conditions: x=1→ $Elim_1$=0.298 eV and x=0→ *Elim*=0 eV.

Effect of changing the limit conditions of the linear correlation between the *Elim* and the *x* values (Model 2): I next evaluate the effect of modifying the limit conditions in the correlation fit, and more particularly the lower region of the fit ($x < 0.05$). At RT, complexes **4-5** possess almost negligible PLQY ($\Phi_{phos} < 0.01$). They are also characterized by the lowest computed *Elim* values (*Elim* < 0.06 eV). These facts point out that these small barriers are extremely accessible at RT. Therefore, for **4-5**, these processes occur in a "barrierless" manner and a new lower limit condition can be proposed, i.e. x=0→ $Elim_4$=0.042 eV (note that this is the computed *Elim* value for **4**), instead of x=0→ *Elim*=0 eV that was used in Model 1. This correlation fit (maintaining the previous upper limit condition) is shown in Fig S2. Complexes with *Elim* values above (below) the upper (lower) limit condition, possess x=1 (x=0).

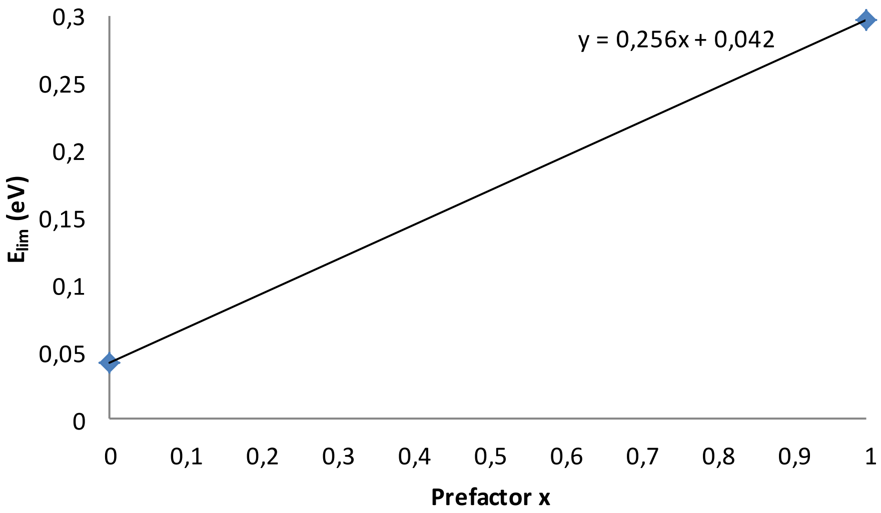

Figure **S2.** Linear correlation between the E_{lim} and x values using as limit conditions: x=1→ $Elim_1$=0.298 eV and x=0→ $Elim_4$=0.042 eV.

Using this correlation fit new prefactor *x(Model 2)* values are obtained (see Table S1). Upon substitution on Eq. (6) new PLQY are obtained and are also tabulated in Table S1.

Table S1. Prefactor *x(Model2)* for 1-6 using the correlation fit of Fig. S2. PLQY obtained with these *x(Model 2)* values.

Complex	Φ_P *(theo, Model2)*	*x (Model2)*
**1**	-	1
**2**	0.87	0.90
**3**	0.55	0.37
**4**	0.00	0.00
**5**	0.04	0.07
**6**	0.52	0.82

As seen in Table S1, with the new prefactor *x(Model 2)* values, very similar theoretical estimators of the PLQY to those listed in Table 1 are obtained. Obviously, since the correlation fit presented in Fig. S2 is more suited to reproduce the lower limit conditions, better comparison with the experimental evidences are obtained for **4-5**. In general, the modification of the limit conditions does not have a great impact on the qualitative pre-screening of phosphors, since the trends in the computed PLQY of the Ir(III) series are maintained, i.e., **2** > **3** > **6** > **4** ≥ **5**, and the model still discerns from highly emissive (**1**-**2**) to non-emissive (**4-5**) or intermediately emissive complexes (**3**,**6**) at RT.

Hyperbolic correlation between the *Elim* and the *x* values (Model 3): I next evaluate the effect of assuming a hyperbolic correlation between the *Elim* and the *x* values. This hyperbolic fit is particularly designed to obtain the expected asymptotic behavior for the lower ($x < 0.05$) and upper ($x > 0.95$) regions of the fit whilst maintaining the predominant linear correlation in the middle region ($0.05 < x < 0.95$). Indeed, this curve is best suited to simulate the drop off of the emission lifetimes of these complexes with increasing temperatures (see e.g. Figures 4-5 of Ref.11). As remarked for Model 2, there is a lower limit *Elim* value ($Elim_4$=0.042 eV), below which the non-radiative pathways are fully operative. Likely, and also experimentally corroborated (since **1-2** possess PLQY values of almost unity at RT), an upper limit *Elim* value ($Elim_2$=0.272 eV) can be defined, above which the non-radiative pathways are mostly quenched. In Model 3, I assume that the limiting barriers for complexes 4 and 2, i.e. $Elim_4$ and $Elim_2$, provide the change from linear to asymptotical tendency of the symmetric sigmoid function, i.e. x=0.05→ $Elim_1$=0.042 eV and x=0.95→ $Elim_4$=0.272 eV. The asymptotes are assigned as x=0→ *Elim*=0 eV and x=1→ *Elim*=0.31 eV and a symmetric condition is assigned at x=0.5→ *Elim*=0.155 eV. Complexes with *Elim* values above the asymptotic limit condition, i.e. *Elim*>0.31 eV, possess x=1. This correlation fit along with its correlation function is shown in Figure S3.

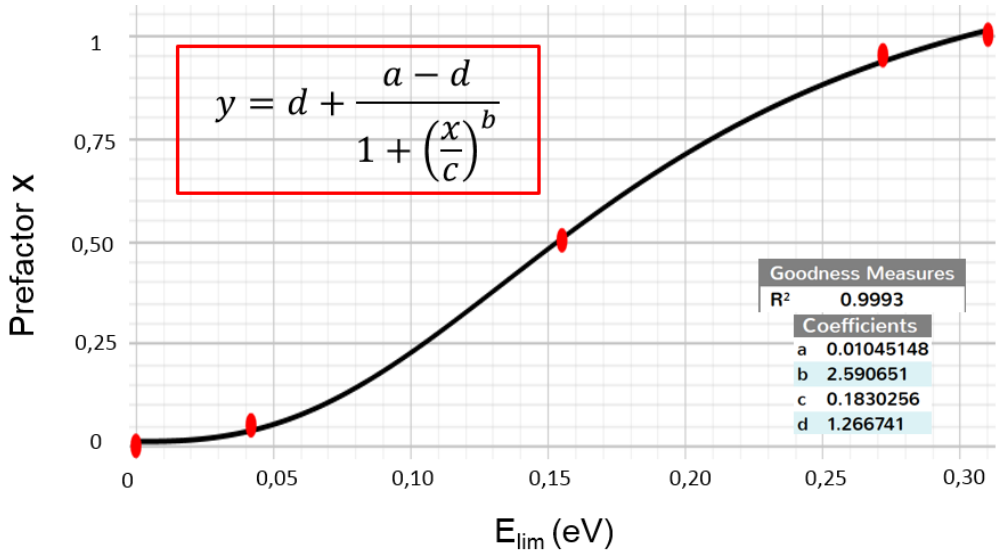

Figure **S3.** Hyperbolic correlation between the E_{lim} and x values. The used correlation function along with its coefficients and goodness measure are also shown.

Using this correlation fit new prefactor *x(Model3)* values are obtained (see Table S2). Upon substitution on Eq. (6) new PLQY are obtained and are also tabulated in Table S2.

Table S2. Prefactor *x(Model3)* for 1-6 using the correlation fit of Fig. S3. PLQY obtained with these *x(Model3)* values.

Complex	*Φ* Φ_P *(theo, Model3)*	*x (Model3)*
**1**	-	1.00
**2**	0.92	0.95
**3**	0.59	0.41
**4**	0.06	0.05
**5**	0.04	0.08
**6**	0.65	0.89

As seen in Table S2, with the new prefactor *x(Model3)* values, similar theoretical estimators of the PLQY to those listed in Table 1 and S1 are obtained. The correlation fit presented in Fig. S3 is best suited to reproduce both the lower and upper limit conditions, since better comparison with the experimental evidences are obtained for the highly emissive **2** complex and the non-emissive **4-5** complexes. Regarding the intermediately emissive **3**,**6** complexes the order in their PLQY is reversed with respect to Models 1 and 2, i.e. **6** > **3**. Still, Model 3 is well suited to discern from highly emissive (**1**-**2**) to non-emissive (**4-5**) or intermediately emissive complexes (**3**,**6**) at RT.

Summary of the correlation models: In Table S3 are listed the results for the prefactor x and PLQY using Models 1-3 along with the experimental values of the PLQY.

Table S3. Summary of the prefactor *x* and PLQY values using the different Model 1-3.

Complex	*x* (Model1)	*x* (Model2)	*x* (Model3)	Φ_P (theo, Model1)	Φ_P (theo, Model2)	Φ_P (theo, Model3)	Φ_P (exp, RT) [a]
**1**	1	1	1	-	-	-	0.97
**2**	0.91	0.90	0.95	0.88	0.87	0.92	0.98
**3**	0.46	0.37	0.41	0.63	0.55	0.59	0.55
**4**	0.14	0.00	0.05	0.16	0.00	0.06	<0.01
**5**	0.20	0.07	0.08	0.11	0.04	0.04	<0.01
**6**	0.85	0.82	0.89	0.57	0.52	0.65	0.37

[a] From Ref. 11.

3. *Choosing a different molecule as a reference.*

Effect of changing the reference molecule in evaluating the PLQY: In the following I evaluate the effect of choosing a different reference molecule. As in the experimental setups, a reference value is needed to compute the PLQY with Eq. (6). In the manuscript this reference value is the experimental $\Phi_{Phos}(298K)$ value of complex **1**, i.e. $\Phi_1(298K) = 0.97$. In the following, I evaluate how the results are affected by choosing a different reference molecule. Towards obtaining maximum sensitivity on the PLQY estimations with Eq. (6), complexes with near the unity of quantum yield are required. Therefore, complex **2** is herein chosen, being the new reference value its experimental PLQY, i.e. $\Phi_2(298K) = 0.98$. Correspondingly Eq. (6) is transformed to

$$\frac{\Phi_X(298K)}{\Phi_2(298K)} = \frac{x\frac{kr_x}{kr_2}}{x\frac{kr_x}{kr_2} + (1-x)\frac{E\lim_x}{E\lim_2}}, \qquad 0 \le x \le 1 \qquad (7).$$

The PLQY values of **1**,**3**-**6** using Models 1-3 with Eq. (7) are presented in Table S4.

Table S4. Computed PLQY values with Models 1-3 using **2** as a reference molecule.

Complex	*Φ_P (theo, Model1)*	*Φ_P (theo, Model2)*	*Φ_P (theo, Model3)*
**1**	0.98	0.98	0.98
**2**	-	-	-
**3**	0.64	0.56	0.60
**4**	0.17	0.00	0.06
**5**	0.11	0.04	0.04
**6**	0.58	0.53	0.66

As seen in Table S4, the effect of choosing a different reference molecule has a smaller effect on the PLQY than the choice of the model to evaluate the PLQY. The new PLQY values are only deviated by ±0.01 from the values obtained using complex **1** as a reference (see Table S3).

4. *Table S5.*

Table S5. Activation barriers (eV) for the temperature-dependent non-radiative channels (see Scheme 1), prefactors *x* and estimated PLQY using Models 1-3 for **7**-**8.**

Complex	E_a	E_b	E_c	E_{lim} [a]	*x(Model 1)*	*x(Model 2)*	*x(Model 3)*	*PLQY (Model1)*	*PLQY (Model 2)*	*PLQY (Model 3)*
**7**	0.211	0.203	0.097	0.211	0.71	0.78	0.75	0.63	0.71	0.68
**8**	0.311	0.218	0.091	0.311	1	1	1	0.97	0.97	0.97

[a] The E_{lim} value usually corresponds to E_a or E_c value, depending on the kinetic scenario.

5. References:

[1] A. D. Becke, *J. Chem. Phys.,* 1993, **98**, 5648.

[2] C. T. Lee, W. T. Yang, R. G. Parr, *Phys. Rev. B.,* 1988, **37**, 785.

[3] D. Andrae, U. Häusermann, M. Dolg, H. Stoll, H. Preuss, *Theor. Chim. Acta,* 1990, **77,** 123.

[4] J. Harvey, M. Aschi, H. Schwarz, W. Koch, *Theor. Chim. Acta,* 1998, **99**, 95.

[5] F. Neese. Orca, an ab initio, DFT and semiempirical SCF-MO package 2.8.0 R2327, University of Bonn: Bonn, Germany, 2011.

[6] Gaussian 09, Revision A.1, M. J. Frisch, G. W. Trucks, H. B. Schlegel, G. E. Scuseria, M. A. Robb, J. R. Cheeseman, G. Scalmani, V. Barone, B. Mennucci, G. A. Petersson, H. Nakatsuji, M. Caricato, X. Li, H. P. Hratchian, A. F. Izmaylov, J. Bloino, G. Zheng, J. L. Sonnenberg, M. Hada, M. Ehara, K. Toyota, R. Fukuda, J. Hasegawa, M. Ishida, T. Nakajima, Y. Honda, O. Kitao, H. Nakai, T. Vreven, J. A. Montgomery, Jr., J. E. Peralta, F. Ogliaro, M. Bearpark, J. J. Heyd, E. Brothers, K. N. Kudin, V. N. Staroverov, R. Kobayashi, J. Normand, K. Raghavachari, A. Rendell, J. C. Burant, S. S. Iyengar, J. Tomasi, M. Cossi, N. Rega, J. M. Millam, M. Klene, J. E. Knox, J. B. Cross, V. Bakken, C. Adamo, J. Jaramillo, R. Gomperts, R. E. Stratmann, O. Yazyev, A. J. Austin, R. Cammi, C. Pomelli, J. W. Ochterski, R. L. Martin, K. Morokuma, V. G. Zakrzewski, G. A. Voth, P. Salvador, J. J. Dannenberg, S. Dapprich, A. D. Daniels, Ö. Farkas, J. B. Foresman, J. V. Ortiz, J. Cioslowski, and D. J. Fox, Gaussian, Inc., Wallingford CT, 2009.

[7] T. Helgaker, H. J. A. Jensen, P. Jorgensen, J. Olsen, K. Ruud, H. Ågren, T. Andersen, K. L. Bak, V. Bakken, O. Christiansen, et. al. Dalton, A Molecular Electronic Structure Program, Release 1.2, 2001

[8] (a) B. Minaev, *J. Phys. Chem. A* 1999, **103**, 7294 (b) O. Rubio-Pons, B. Minaev, O. Loboda, H. Ågren, *Theor. Chem. Acc.* 2005, **113**, 15. (c) O. Rubio-Pons, O. Loboda, B. Minaev, B. Schimmelpfennig, O. Vahtras, H. Ågren, *Mol. Phys.* 2003, **101**, 2103.

[9] (a) M. Douglas, N. M. Kroll, *Ann. Phys.* 1974, **82**, 89 (b) B. A. Hess, *Phys. Rev. A* 1986, **33**, 3742.

[10] S. Koseki, M. Schmidt, M. Gordon, *J. Phys. Chem.* 1990, **96**, 10678.

[11] T. Sajoto, P. I. Djurovich, A. B. Tamayo, J. Oxgaard, W. A. Goddard III, M. E. Thompson, *J. Am. Chem. Soc.*, 2009, **131**, 9813.
